# Understanding the participation outcomes for persons with disability when partnered with assistance dogs: A scoping review

**DOI:** 10.1111/1440-1630.12801

**Published:** 2022-04-25

**Authors:** Nicola Futeran, Lynette Mackenzie, Sarah Wilkes‐Gillan, Claire Dickson

**Affiliations:** ^1^ Discipline of Occupational Therapy, School of Health Sciences, Faculty of Medicine and Health The University of Sydney Sydney New South Wales Australia; ^2^ Occupational Therapy Assistance Dogs Australia Engadine New South Wales Australia

**Keywords:** assistance dog, disabled persons, dogs, participation, service dog

## Abstract

**Introduction:**

Assistance dogs are trained to support persons living with disability and mitigate limitations that hinder their participation in everyday activities. Despite participation being a frequent challenge for people with disabilities, evidence linking assistance dog provision to improved participation outcomes is underdeveloped. This scoping review aimed to improve understanding by mapping the participation outcomes claimed in research on assistance dogs using the International Classification of Functioning (ICF), Disability and Health framework.

**Methods:**

Using the Arksey and O′Malley's six‐step framework, this scoping review searched six databases. Data were collected, mapped and summarised in accordance with the domains outlined in the ICF.

**Results:**

In total, 38 studies across 41 papers met the inclusion criteria. Included studies investigated assistance dogs who were partnered with people living with physical disabilities, mental illness, autism and chronic conditions that require alerting (e.g., epilepsy and diabetes). Mapping of participation outcomes suggested that assistance dogs can have a positive impact on participation in many areas of daily life.

**Conclusion:**

Findings can assist practitioners, funders and policymakers to recognise the value of assistance dogs as a support for people with disability. However, further research is needed to address limitations regarding study designs, for example, the outcome measures used.

**Key Points for Occupational Therapy**

- More attention is being given to the potential benefits of assistance dogs for a range of occupational therapy client groups such as children with Autism, persons with physical disability and mental health issues.
- The evidence supporting assistance dog placement is summarised in this scoping review and provides evidence that can be used to support application for funding support for assistance dogs.
- Occupational therapists have a role in promoting participation and recommending assistive technology (including assistance dogs).

## 1 INTRODUCTION

Participation is defined by the World Health Organisation's (WHO) International Classification of Functioning, Disability and Health (ICF) as involvement in a life area (WHO, 2001) and is a core concept within occupational therapy practice (Vessby & Kjellberg, 2010). Persons with disability are often faced with challenges to achieving full participation (Hammel et al., 2008). To address participation concerns, assistive technologies and supports are commonly implemented (Lenker et al., 2013; Ripat & Woodgate, 2012). Provision of assistance dogs is an example of an emerging support intervention that aims to address the unique participation needs of persons with disability.

The United Nations has recognised participation as a basic human right (United Nations, 2006) and the ICF considers activities and participation as central constructs of health, alongside body systems and structures influenced by the environment and personal factors (WHO, 2001). Participation facilitates skill acquisition, meaning and purpose in life as well as connection with society, and participation restrictions can negatively influence psychological, physical, development and well‐being, ultimately impacting quality of life (Chao, 2014; Law, 2002; Martin Ginis et al., 2017). The ICF also considers that participation is not only predictive of an individual's disability but also how they function in their context (Hammel et al., 2015; Rimmer, 2006). Participation has consequently been identified as one of the most highly valued rehabilitation outcomes and a fundamental goal of disability service providers (Magasi et al., 2009). Persons with disability may face barriers to participation, and the personalised, ongoing support of an assistance dog may address some of these barriers.

Assistance dogs, as distinct from therapy dogs, reside with persons with disability and have specialised training to mitigate any limitations (Assistance Dogs International [ADI], 2019). Assistance dogs have been trained to support individuals with disabilities other than vision or hearing deficits, such as physical disabilities, mental illness, autism, diabetes or epilepsy, and in the United States, they have public access rights, facilitating improved participation outcomes in the home and in the community (ADI, 2019). Such laws in Australia are more varied. The dog is cared for by the individual with a disability (Parenti et al., 2013) creating a reciprocal partnership.

Although the outcomes associated with assistance dog partnerships may overlap with the psychological, social, physical and developmental benefits of pets (Christian et al., 2013; McNicholas & Collis, 2000; Purewal et al., 2017; Wood et al., 2005), the level of training and lengthy matching process differentiate assistance dogs. Assistance dogs are also distinguished from pets and emotional support animals (Schoenfeld‐Tacher et al., 2017). In this study, the definition of an assistance dog will align with ADI.

For people with physical disabilities, assistance dogs can be trained to complete physically demanding tasks including, switching lights on and off, opening doors, and retrieving dropped items (Crowe et al., 2014). Assistance dogs can provide limited physical support for ambulation and transferring (Blanchet et al., 2013). Challenges to socialising, particularly for people with autism, can be eased by the friendly nature and companionship of an assistance dog that can sense anxiety and distress and take steps to calm (Burrows et al., 2008; Gilbey & Tani, 2015). For people living with mental illness, an assistance dog can be trained to recognise concerning behaviours and comfort or distract the individual (Lloyd et al., 2019). Assistance dogs can also be trained to detect and respond to medical conditions like hypoglycaemic episodes for people with diabetes or seizures for people with epilepsy (Catala et al., 2018; Lippi & Plebani, 2019).

However, there are limitations to the way research can be done. The lengthy process to allocate and place assistance dogs has led to small sample sizes and the absence of controls, and many studies are also reliant on self or parental report. Participation outcomes are not commonly linked to assistance dogs in studies. Understanding the participation outcomes associated with assistance dogs is important for service providers, health professionals and funding bodies who aim to improve participation outcomes for persons with disabilities (Heinemann et al., 2013). However, previous reviews have not focused on the participation outcomes of assistance dog partnership. Sachs‐Ericsson et al. (2002) did consider participation outcomes, but the review only included assistance dogs for persons with hearing or physical impairments. More recent reviews considered participation outcomes but were limited to a single disability group (van Houtert et al., 2018; Winkle et al., 2012).

Therefore, this study aimed to conduct a scoping review of the participation outcomes of assistance dog partnerships. This aim used the ICF framework to map the participation outcomes from assistance dog studies. The following research questions guided the review:

1. What are the participation outcomes for persons living with disability when partnered with an assistance dog?
2. What are the contextual factors that can influence the participation outcomes for persons with disability?
3. What types of available research describe outcomes of the partnership between persons with disability and assistance dogs?

## 2 METHODS

A scoping review study design using the six‐step method outlined by Arksey and O'Malley (2005) was selected to map the breadth of the role of assistance dogs and consequent participation outcomes. A scoping review can incorporate multiple study types, acknowledging the variety of quantitative and qualitative studies investigating the assistance dog partnership (Colquhoun et al., 2014; Levac et al., 2010; Munn et al., 2018). The Preferred Reporting Items for Systematic Reviews and Meta‐Analyses (PRISMA) extension for scoping reviews also guided the methodology and reporting of this study (Tricco et al., 2018).

### 2.1 Eligibility criteria

Included studies were peer‐reviewed and investigated the partnership between people with disabilities and their assistance dogs. Studies were published in English between 1 January 2000 and 31 January 2020 inclusive.

Aligning with definitions provided by ADI (2019), an assistance dog was defined as follows: (1) having completed certified training and passed a public access test; (2) providing ongoing support to a single person with a disability; and (3) having a primary carer who was either a person living with a disability or their designated guardian. Studies of guide dogs and dogs for those with hearing impairments were excluded as the evidence base for these is well‐established.

To be included in the review, study outcomes needed to relate to participation and be consistent with the *activity and participation* domains in the ICF (WHO, 2001). Studies were included if participation outcomes related to the person for whom the assistance dog was placed or for their family. Participation outcomes could be primary or secondary objectives of the study. Studies focusing only on psychological or physiological outcomes with no task‐related impact (e.g., upper limb effort or seizure frequency), or studies focusing on the assistance dog alone (e.g., welfare, training, or breeding), were excluded.

Publications without full‐text availability, theses, dissertations, editorials, opinion pieces and conference papers were excluded. The papers included in systematic and scoping reviews were included if they met the inclusion criteria.

### 2.2 Information sources and search

To identify relevant studies, six electronic databases were searched: Medline, Allied Health Literature (CINAHL), Scopus, PsychINFO, PsycARTICLES and Cochrane CENTRAL. The search strategies were developed and refined by the first and second authors in consultation with an expert Librarian from the University of Sydney. Search strategies included four key search terms: assistance dog, assistance animal, service dog and service animal. The final search strategy for Medline is available on request. The final search results from all databases were downloaded into endnote, and duplicates were removed.

To comprehensively search the literature, the electronic database search was supplemented by hand‐searching HABRI Central (Human‐Animal Bond Research Initiative) and Human‐animal Interaction Bulletin. Reference lists from review studies investigating assistance dogs and from included papers were also screened to retrieve any relevant studies.

### 2.3 Study selection

A screening checklist, aligning with the eligibility criteria, was created to increase consistency in the selection of eligible studies. After the first author (NF) screened 10% of the retrieved titles and abstracts, a discussion between NF and LM led to refinement and then 100% agreement on the final checklist.

**FIGURE 1:**
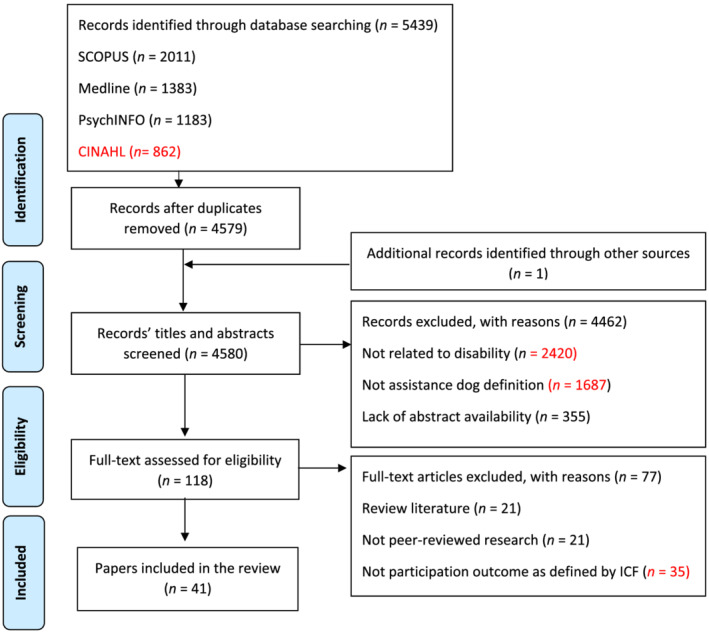
Preferred Reporting Items for Systematic Reviews and Meta‐Analyses (PRISMA) flow chart

The selection of eligible studies was conducted by the first and second authors in a two‐stage screening process. First, all titles and abstracts were screened for relevance by NF, and all were cross‐checked by LM. Titles and abstracts that did not meet the eligibility criteria were excluded. Second, studies with the potential to meet the eligibility criteria were independently screened at a full‐text level by NF and LM. Any disagreements regarding selection were resolved through discussion between authors and by involving SWG who mediated until full consensus was reached. A PRISMA flow diagram detailing the selection process was created (Moher et al., 2009). See Figure 1.

### 2.4 Data charting process and data items

A data‐charting form was designed by NF to determine the relevant information to extract from the included studies, guided by recommendations from the Joanna Briggs Institute (Peters et al., 2020). The form recorded the study, participant, and assistance dog characteristics, outcome measures used, and the main outcomes reported in quantitative studies and the main themes documented in qualitative studies. Quantitative studies were classified by the National Health and Medical Research Council (NH&MRC) levels of evidence to indicate the strength of the study designs and any risk of potential bias (NH&MRC, 2009).

The first author (NF) charted the data into the form. The second author (LM) cross‐checked all the extracted data to verify the charting accuracy. Disagreements were resolved through discussion. Where additional detail was required, the first author (NF) contacted the study authors.

After data were extracted, the studies were organised into groups according to participant conditions. Groups included participants living with autism, physical disabilities, mental illness and disabilities requiring alert (i.e., people living with diabetes or experiencing seizures). A fifth group, labelled *combination*, referred to studies including participants with a range of disabilities. However, if these studies included distinct results for each type of disability, then the data were separated and charted in the relevant disability group.

### 2.5 Synthesis of results

Data from included studies were analysed in relation to the three research questions for the review. The first author (NF) completed the analysis process, and the findings were discussed with all authors. To determine the overall characteristics and limitations of the current assistance dog literature, descriptive summary statistics were derived using Excel.

To determine the participation outcomes, included study outcomes were mapped against the nine ICF *activity and participation* domains (WHO, 2001). Mapping was facilitated by coding relevant findings according to the ICF domains. The assessment items of the outcome measures used in quantitative studies were also coded against the domains. Codes were classified as a positive, negative, significant, non‐significant or a mixed outcome. The category mixed outcome referred to studies that had a positive and negative outcome or significant and non‐significant outcomes. Codes were initially allocated by the first author (NF) and cross‐checked by the other authors.

To determine contextual factors that could influence the participation outcomes of the partnership, relevant findings were mapped against the five ICF *environmental factors* domains using the same coding process (WHO, 2001). These domains include the following: *products and technology*; *natural environment and human‐made changes to the environment*; *support and relationships*; *attitudes*; and *services, systems, and policies*. If factors were related directly to the participant's life (e.g., past life events, character style and health conditions), the factor was coded as a *personal factor*. *Personal factors* are not categorised into domains by the ICF (WHO, 2001).

The ICF framework includes additional categories under each domain. The categories are in a hierarchical structure, where the lower level categories provide a more detailed explanation about the domain (WHO, 2001). The categories were referred to during the coding process to promote accuracy. For instance, leisure as a participation outcome would be mapped to Recreation and Leisure (d920), and the domain of Community, Social and Civic Life.

## 3 RESULTS

### 3.1 Study selection

The search strategy retrieved 5439 records. Only one record was added after hand‐searching, and 860 duplicates were removed. The remaining 4580 titles and abstracts were screened by the first author (NF) and cross‐checked by the second author (LM). Authors disagreed on the eligibility of 81 records (1.8%) to proceed to full‐text screening. A total of 38 studies across 41 papers met the eligibility criteria and were included in the review; see Figure 1.

### 3.2 Characteristics of sources of evidence

Participant, study and assistance dog characteristics are described in Table S1. The data are separated according to participant disability.

#### 3.2.1 Participant characteristics

Across studies, participants included people living with physical disability (*n* = 16), mental illness, including psychiatric diagnoses and traumatic brain injury (*n* = 12), autism (*n* = 5), and epilepsy or diabetes (*n* = 2). One study contained extractable data concerning people with physical disabilities and diabetes (Lundqvist et al., 2018). The remaining four studies (five papers) were charted under the combination group as the studies investigated participants with multiple limitations. Overall, studies investigated outcomes for the person living with disability (*n* = 32), the family (*n* = 2) or both (*n* = 4).

The 38 studies included a total of 1956 persons living with disability, ranging between 1 and 199 participants per study. Of studies that reported age and gender, the mean age of participants was 34 years (SD = 13.2, range of means = 6.7–44.7), and 56.2% were male. In studies where individuals were diagnosed with autism, the mean age was 6.89 years (SD = 0.8, range of means = 6.7–8.3), and most participants were male (85.78%). Ethnicity was only reported in seven studies.

The most common diagnosis indicated in studies for people living with physical disability was a spinal cord injury (*n* = 11) followed by cerebral palsy (*n* = 5). For people living with mental illness, 10 studies included veterans with post‐traumatic stress disorder (PTSD). For studies investigating people living with autism, their reported diagnosis was autism spectrum disorder or Asperger syndrome or pervasive developmental disorder—not otherwise specified, aligning with DSM‐V (American Psychiatric Association, 2013).

#### 3.2.2 Study characteristics

Most papers were published in the last 5 years (2015–2020; *n* = 25; 61.0%), and almost all were published within the last 15 years (2005–2020; *n* = 40; 97.6%). Papers originated from seven countries with the majority conducted in the United States (*n* = 15; 36.6%) and Canada (*n* = 14; 34.1%). The 38 studies included quantitative (*n* = 21), qualitative (*n* = 16) and mixed‐methods (*n* = 1) designs. According to the NHMRC hierarchy of evidence, most quantitative studies were classified as level IV (*n* = 15) or level III‐3 (*n* = 5), and only one non‐randomised control trial was level III‐2. Data collection methods included the use of standardised measures (*n* = 17) and/or assessment tools designed by the authors of the study (*n* = 12).

Qualitative approaches composed of case study (*n* = 6), phenomenology (*n* = 3), ethnography (*n* = 2), exploratory (*n* = 2) and descriptive (*n* = 1) designs. There were two studies that did not specify the qualitative approach. Data collection methods included interviews (*n* = 15), observations (*n* = 4) and focus groups (*n* = 2).

#### 3.2.3 Assistance dog characteristics

Across the studies, assistance dogs were referred to using the following terminology: service dog (*n* = 30; 78.9%), assistance dog (*n* = 4; 10.5%), seizure alert dog (*n* = 1; 2.6%) or a combination of these terms (*n* = 3; 7.9%). The assistance dog was placed prior to the commencement of the study (*n* = 29), as a part of the study (*n* = 7), or both (*n* = 2). Participants placed with assistance dogs before the study commenced had lived with an assistance dog for an average of 3.4 years (SD = 3.4, range of means = 1–5).

Length of training for the individual with their assistance dog varied. Some organisations required the individual and their dog to attend an intensive program running for 5–21 days, while others required weekly training for up to 18 months. The specific aim of the placement of the assistance dog was reported in 14 studies. Reasons for placement commonly included mobility assistance, functional assistance, management of mental health or promotion of safety.

### 3.3 Identification of participation outcomes

Table 1 outlines the included studies mapped to the *activity and participation* domains outlined by the ICF (WHO, 2001). All nine domains were addressed across the included studies. The most common domains mapped across all disability groups were *general tasks and demands* and *interpersonal interactions and relationships* (*n* = 35; 92.1%). Other frequently addressed domains included *community*, *social and civic life* (*n* = 28; 73.7%), *major life areas* (*n* = 26; 68.4%), *self‐care* (*n* = 23; 60.5%), *domestic life* (*n* = 23; 60.5%) and *mobility* (*n* = 22; 57.9%). The frequency of these domains differed between population groups.

**TABLE 1:**
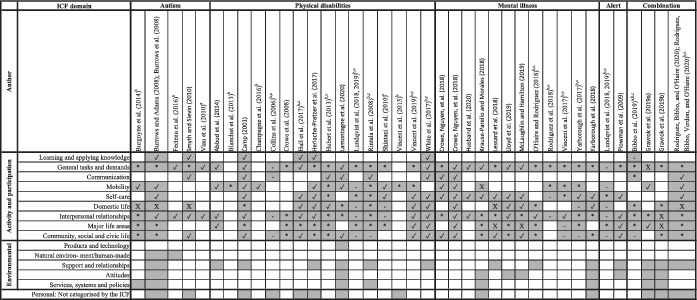
Mapping of ICF activity and participation domains, environmental factors domains and personal factors

*Note*: Shaded = *activity and participation* domain, *environmental factors* domain or *personal factor* addressed in the corresponding study; ✓ = positive or significant outcomes *p* < 0.05; X = negative or adverse significant outcomes *p* < 0.05; * = mixed outcomes (study includes positive and negative outcomes or significant and non‐significant outcomes); ‐ = non‐significant outcomes *p* >0.05.

Abbreviation: ICF, International Classification of Functioning.

^a^ Outcome measure for quantitative study was not able to be accessed by review authors.

^b^ Outcome measures or subscales covered more than one *activity and participation* domain, and it was not possible to separate.

^c^ More than one outcome measure or subscale contributed to the same domain.

The *activity and participation* domains mapped were classified as positive or significant (47.6%), mixed (38.1%) or negative or non‐significant (14.3%). The domain that had the most studies classified as positive or significant was *self‐care* (*n* = 18) followed by *interpersonal interactions and relationships* (*n* = 16). The *general task and demands* domain had the greatest number of studies with mixed outcomes (*n* = 21), and the *domestic life* domain had the greatest number of studies with negative or non‐significant outcomes (*n* = 8).

The main participation outcomes that fell under each domain, with relevant examples, are provided in Table 2. Across domains, this totalled 21 outcomes. Almost all the participation outcomes were mapped across all disability groups (*n* = 18, 85.7%), and therefore outcomes were tabulated together. The most common outcomes included the following: psychological demands of tasks (*n* = 27), general task participation (*n* = 19), human–human relationships (*n* = 25), animal–human relationships (*n* = 17), social interactions (*n* = 17) and getting around (*n* = 16). All participation outcomes related to the person living with a disability, while 14 of the outcomes also related to the family members.

**TABLE 2:** Participation outcomes and examples

Participation outcome	Positive/significant outcome examples	Mixed outcome examples	Negative/non‐significant outcome examples
General tasks and demands			
Psychological demands of tasks	Increase in responsibility, providing a sense of worth and purpose. P: Burgoyne et al. (2014), Crowe, Sánchez, et al. (2018), Lloyd et al. (2019), Yarborough et al. (2018). Increase feelings of safety and security, improving confidence. P: Lloyd et al. (2019), Crowe, Sánchez, et al. (2018), Lamontagne et al. (2020), Plowman et al. (2009). Improved ability to deal with lifes stressors. P: Lloyd et al. (2019), Crowe, Sánchez, et al. (2018), Husband et al. (2020). S: Viau et al. (2010).	Dealing with symptoms of mental illness which impact life participation. P: Lessard et al. (2018), Lloyd et al. (2019), Yarborough et al. (2017), Yarborough et al. (2018). S: O'Haire and Rodriguez (2018), Rodriguez et al. (2018), NS: Collins et al. (2006), Rintala et al. (2008). MS: Shintani et al. (2010), Vincent et al. (2017), Yarborough et al. (2017). Family: Coping with lifes stressors. P: Burgoyne et al. (2014), Plowman et al. (2009), Smyth and Slevin (2010). S: Burgoyne et al. (2014), Fecteau et al. (2016), MS: Bibbo et al. (2019).	
Task participation	Reduced paid assistance. S: Rintala et al. (2008). Improved task performance. P: Herlache‐Pretzer et al. (2017), Camp (2001), Rintala et al. (2008), Krause‐Parello et al. (2016). Parent: improved management of child and fulfilment of parental duties.	Independence in daily tasks. NS: Rintala et al. (2008), Vincent et al. (2019). S: Hall et al. (2017). P: Burrows et al. (2008), Camp (2001), Crowe, Sánchez, et al. (2018), Herlache‐Pretzer et al. (2017), Lloyd et al. (2019), Plowman et al. (2009), Rintala et al. (2008). Number of tasks completed. MS: Yarborough et al. (2017). NS: Rintala et al. (2008). Family: Daily activity participation. P: Burgoyne et al. (2014). NS: Bibbo et al. (2019).	Additional workload (dog training requirements). N: Herlache‐Pretzer et al. (2017), Lessard et al. (2018), Plowman et al. (2009), Yarborough et al. (2018). Same for family. N: Burrows et al. (2008).
Carrying out daily routine	Increase in routine engagement. P: Crowe et al. (2014), Crowe, Sánchez, et al. (2018), Herlache‐Pretzer et al. (2017).	Bedtime routine and sleep quality. P: Burrows et al. (2008), Husband et al. (2020), Krause‐Parello and Morales (2018), Lessard et al. (2018), Lloyd et al. (2019), Smyth and Slevin (2010). S: Vincent et al. (2017). NS: Yarborough et al. (2017). MS: Rodriguez et al. (2018). Family: Sleep quality. P: Burrows et al. (2008). NS: Bibbo et al. (2019).	Unwanted adaption to routine.
Managing own activity level	Reduced time, energy and effort to complete tasks. P: Camp (2001), Crowe et al. (2014), Herlache‐Pretzer et al. (2017).	Vitality. NS: Lundqvist et al. (2018), Shintani et al. (2010). MS: Vincent et al. (2019).	
Interpersonal interactions and relationships			
Human‐human relationship	Re‐established or new relationships. P: Crowe, Sánchez, et al. (2018), Lessard et al. (2018), Yarborough et al. (2018). Improved relationships with family. P: Abbud et al. (2014), Burgoyne et al. (2014), Crowe, Sánchez, et al. (2018), Krause‐Parello et al. (2016), Lloyd et al. (2019). S: Burgoyne et al. (2014) Fecteau et al. (2016), Hall et al. (2017), O'Haire and Rodriguez (2018). Improved relationship with friends. P: Plowman et al. (2009), Increase in the number of social opportunities and interactions. P: Abbud et al. (2014), Camp (2001), Crowe, Sánchez, et al. (2018), Herlache‐Pretzer et al. (2017), Lamontagne et al. (2020), Lessard et al. (2018), Plowman et al. (2009).	Social functioning. S: Hall et al. (2017), Hubert et al. (2013), O'Haire and Rodriguez (2018). NS: Shintani et al. (2010). MS: Yarborough et al. (2017). Interactions with public/strangers. P: Abbud et al. (2014), Crowe, Sánchez (2018), Lessard et al. (2018). S: Burgoyne et al. (2014), O'Haire and Rodriguez (2018). N: Herlache‐Pretzer et al. (2017), Lamontagne et al. (2020), Lloyd et al. (2019), Yarborough et al. (2018). Interactions with family. P: Burrows et al. (2008), Smyth and Slevin (2010). N: Burrows et al. (2008). Family cohesion. P: Smyth and Slevin (2010). MS: Bibbo et al. (2019).	Loss of relationships with others due to issues with accepting the dog or allergy issues.
Animal‐human relationship	Improved sense of companionship. P: Burgoyne et al. (2014), Burrows et al. (2008), Camp (2001), Crowe, Sánchez, et al. (2018), Herlache‐Pretzer et al. (2017), Husband et al. (2020), Lamontagne et al. (2020), Lessard et al. (2018), Plowman et al. (2009), Smyth and Slevin (2010). Correlation between attachment to assistance dog and quality of life. S: White et al. (2017).	Sense of isolation and loneliness. P: Lessard et al. (2018), Crowe, Sánchez, et al. (2018), Husband et al. (2020). S: O'Haire and Rodriguez (2018). NS: Collins et al. (2006). Family: Sense of companionship. P: Burrows et al. (2008), Smyth and Slevin (2010). NS: Bibbo et al. (2019).	Stress regarding death, retirement, or separation from dog. N: Burgoyne et al. (2014).
Social skills	Improved behaviour. P: Smyth and Slevin (2010). S: Fecteau et al. (2016), Viau et al. (2010). Improved comfort and confidence in social situations. P: Abbud et al. (2014), Camp (2001), Lessard et al. (2018), Lloyd et al. (2019), Yarborough et al. (2018). Improved social skills. P: Crowe, Sánchez, et al. (2018).		
Community, social and civic life			
Community activity participation	Increase in the number of community activities and time in community. P: Burgoyne et al. (2014), Camp (2001), Crowe, Sánchez, et al. (2018), Herlache‐Pretzer et al. (2017), Lamontagne et al. (2020), Lloyd et al. (2019), Plowman et al. (2009), Yarborough et al. (2018). Increase in independence in the community. P: Burgoyne et al. (2014), Burrows et al. (2008), Smyth and Slevin (2010).	Social integration in community. P: Burgoyne et al. (2014), Crowe, Sánchez, et al. (2018), Lloyd et al. (2019). NS: Collins et al. (2006).	Longer to complete community tasks with dog. N: Lloyd et al. (2019). Same for family. N: Burrows et al. (2008).
Recreation and leisure	Increased involvement in leisure activities P: Burgoyne et al. (2014), Camp (2001), Crowe, Sánchez, et al. (2018), Lessard et al. (2018). S: Hall et al. (2017). Same for family. P: Burrows et al. (2008), Smyth and Slevin (2010).	Increased participation in travel but difficulty planning travel to accommodate dog. P: Burrows et al. (2008), Lloyd et al. (2019), Smyth and Slevin (2010), Herlache‐Pretzer et al. (2017). N: Burgoyne et al. (2014), Burrows et al. (2008).	
Well‐being in the community	Increased safety. P: Burgoyne et al. (2014), Burrows et al. (2008), Herlache‐Pretzer et al. (2017), Lessard et al. (2018), Lloyd et al. (2019), Plowman et al. (2009), Smyth and Slevin (2010). S: Burgoyne et al. (2014). Improved confidence in the community. P: Camp (2001), Crowe, Sánchez, et al. (2018), Herlache‐Pretzer et al. (2017), Lessard et al. (2018).		Stressful managing dog the community. N: Yarborough et al. (2018).
Major life areas			
Work	Improved performance/productivity of work‐related tasks. P: Crowe et al. (2014), Crowe, Sánchez, et al. (2018), Herlache‐Pretzer et al. (2017). S: Hall et al. (2017), Lundqvist et al. (2018), O'Haire and Rodriguez (2018). Improvement in work satisfaction. S: Vincent et al. (2019).		Employment status NS: O'Haire and Rodriguez (2018) Family: Work/school function. NS: Bibbo et al. (2019).
Education	Improved school experience. P: Burrows and Adams (2008), Plowman et al. (2009). Improved access to university. P: Camp (2001), Crowe, Sánchez, et al. (2018), Lloyd et al. (2019).		
Financial		Financial burden of vet care and maintenance but benefits can outweigh cost. N: Burgoyne et al. (2014), Burrows et al. (2008), Camp (2001), Lessard et al. (2018), Rodriguez et al. (2018). P: Herlache‐Pretzer et al. (2017), Lundqvist et al. (2018).	Family: Financial burden. N: Burgoyne et al. (2014).
Self‐care			
Maintaining health	Increase in physical activity and fitness. P: Camp (2001), Yarborough et al. (2018). S: White et al. (2017). Improved management of medication through retrieval or reminders. P: Camp (2001), Crowe et al. (2014), Lloyd et al. (2019). Reduced or stabilised prescribed medication. P: Husband et al. (2020), Lessard et al. (2018), Lloyd et al. (2019), Yarborough et al. (2018). Improved outcomes related to health care services. P: Crowe et al. (2014), Lessard et al. (2018), Lloyd et al. (2019). Reduced suicidal ideations or attempts. P: Lloyd et al. (2019), Yarborough et al. (2018).	Reduction in negative behaviours effecting health reported: Self‐medication. P: Crowe, Sánchez, et al. (2018). Use of illicit substances. P: Husband et al. (2020), Krause‐Parello et al. (2016). Overuse of substances. S: Rodriguez et al. (2018), NS: Vincent et al. (2017).	Family: Physical function. NS: Bibbo et al. (2019).
Managing pain	Improved management of pain.	Pain decreased. S: Hubert et al. (2013), Vincent et al. (2019). NS: Lundqvist et al. (2018), O'Haire and Rodriguez (2018), Shintani et al. (2010).	
Personal care tasks	Improved ability to complete self‐care related tasks P: Burrows et al. (2008), Herlache‐Pretzer et al. (2017), Plowman et al. (2009).		
Domestic life			
Domestic activities	Increased participation in community & household domestic tasks P: Camp (2001), Crowe, Sánchez, et al. (2018), Herlache‐Pretzer et al. (2017), Lamontagne et al. (2020), Lloyd et al. (2019). S: Hall et al. (2017).	Increased domestic responsibility related to care of the dog P: Yarborough et al. (2017), N: Camp (2001), Crowe, Sánchez (2019), Lamontagne et al. (2020).	Family: Increase in domestic responsibility. N: Burgoyne et al. (2014), Burrows et al. (2008), Smyth and Slevin (2010).
Mobility			
Getting around	Improved transferring. P: Camp (2001), Herlache‐Pretzer et al. (2017). Improved experience using public transport or cars P: Burrows et al. (2008), Crowe, Sánchez, et al. (2018). Reduced falls risk. P: Herlache‐Pretzer (2018), Lamontagne et al. (2020). Use of stairs P: Herlache‐Pretzer et al. (2017), S: Blanchet et al. (2013).	Wheelchair skills and mobility. P: Camp (2001), S: Blanchet et al. (2013), Vincent et al. (2015), Champagne et al. (2016). MS: Hubert et al. (2013), Vincent et al. (2019). Use of wheelchair on a slope and threshold. MS: Vincent et al. (2015), Vincent et al. (2019). Improved mobility P: Burgoyne et al. (2014), Burrows et al. (2008), Camp (2001). NS: Rintala et al. (2008), Vincent et al. (2017).	Use of stairs descending. NS: Vincent et al. (2015).
Retrieving items	Improved ability to retrieve, lift or carry items P: Camp (2001), Crowe et al. (2014), Herlache‐Pretzer et al. (2017), Lamontagne et al. (2020)		Ability to reach items. NS: Vincent et al. (2015).
Communication			
Ability to communicate	Improved comfort and confidence during conversations. P: Camp (2001), Crowe, Sánchez, et al. (2018), Smyth and Slevin (2010). Improved communication in a medical emergency P: Lamontagne et al. (2020), Rintala et al. (2008).		Family: Communication. NS: Bibbo et al. (2019).
Learning and applying knowledge			
Skill acquisition	Developed motor skills P: Burrows et al. (2008), Smyth and Slevin (2010), safety skills P: Smyth and Slevin (2010) and advocacy skills P: Burrows et al. (2008), Camp (2001), Herlache‐Pretzer et al. (2017). Increase in knowledge. S: Hall et al. (2017).		Family: Cognitive function. NS: Bibbo et al. (2019).

Abbreviations: MS, participation outcome included both significant and non‐significant results; N, negative reported outcome; NS, non‐significant outcome; P, positive reported outcome; S, significant outcome.

### 3.4 Identification of factors influencing participation outcomes

Table 1 provides a list of the included studies mapped against *environmental factors* domains and *personal factors*. The mapping outlines the contextual factors that were reported to impact the participation outcomes of the partnership. Overall, 24 studies (63.2%) referred to a factor impacting the partnership's outcomes.

#### 3.4.1 Environmental factors

Environmental factors influencing the success of the partnership were mapped in 20 studies (52.6%). The most common *environmental factor*s domain was *supports and relationships* (*n* = 15; 39.5%). Potential supports in the social environment included family, friends, carers, community members, health professionals and the government. The social environment was reported to influence the role of the assistance dog in daily tasks, relationships, the community, work, school and health‐care services. The dog itself (e.g., health issues, behaviours and time taken to adjust after training) and the nature of the partnership (e.g., level of attachment and length of partnership) were reported to impact participation outcomes. Another common *environmental factors* domain mapped was *attitudes* (*n* = 9; 23.7%) as societal attitudes to assistance dogs impacted the community and social participation of individuals. Specifically, negative attitudes or lack of understanding about the partnership led to unwanted public attention or questioning, impacting public access with the dog.

Factors mapped under the *services*, *systems and policies* (*n* = 8; 21.1%) domain included the processes of assistance dog‐training organisations and the policies of community organisations such as community‐day programs, schools and health‐care services. Organisations that put in additional effort to create policies and systems to support the partnership were associated with better outcomes. For the *natural environment and human‐made changes to environment* domain, seasonality was considered to influence the partnership's outcomes as winter months meant people were less inclined to venture out into the public with their dog (*n* = 2; 5.3%). For the *products and technology* domain, the home living space was mentioned as a factor relating to partnership success as it needed to accommodate a dog (*n* = 1, 2.6%). However, these domains were mapped infrequently.

#### 3.4.2 Personal factors

Personal factors were mapped in 13 studies (34.2%). The health status of the individual living with disability was reported to impact the outcomes of the partnership (*n* = 8). This included the number of hospital admissions, fluctuation or complication of health conditions, cognition, ability to communicate, level of physical functioning, and mental health status. Psychological characteristics (*n* = 6) were also reported as a main factor that could enhance or detract from the partnership's outcomes. They included flexibility, motivation, confidence, self‐belief and maturity. Additionally, past experiences with animals, gender and marital status were occasionally stated as a factor influencing the dog's role.

## 4 DISCUSSION

This scoping review aimed to identify the participation outcomes impacted by assistance dog partnerships. The review used the globally recognised ICF framework to guide data mapping of the 38 included studies. Mapping suggested there is potential for assistance dogs to impact a wide range of daily‐life areas.

### 4.1 Participation outcomes impacted by assistance dogs

Mapped outcomes were classified as positive or negative, highlighting the value of the partnership. The positive outcomes are comparable with other disability interventions endorsed by health professionals (Rimmer, 2006). All the ICF *activity and participation* domains were mapped across the included studies, indicating that assistance dogs have the potential to impact across many areas of daily life. The *interpersonal interactions and relationships*, *general tasks and demands*, *mobility*, *self‐care*, *domestic life*, *major life areas*, and *community*, *social and civic life* domains were all mapped across studies, irrespective of diagnosis. This adds to current understanding of assistance dogs as many previous reviews only investigated participants with a specific disability (Catala et al., 2018; van Houtert et al., 2018; Winkle et al., 2012). The broad range of participation outcomes highlights the flexibility of the assistance dog partnership that is important as individuals have diverse participation goals and priorities irrespective of diagnosis (Magasi et al., 2009; Martin Ginis et al., 2017). Therefore, flexible interventions, such as assistance dogs, can support a range of participation outcomes. One study suggested that a potential reason for the findings is the adaptable nature of the dog and their individualised training that enables a positive but diverse impact (Heinemann et al., 2013).

Another main finding was in line with previous literature that suggested that social and psychological outcomes were two of the most common benefits of human–dog interactions (Fine & Weaver, 2018). These outcomes fell under the two most frequently mapped domains, *interpersonal interactions and relationships* and *general tasks and demands*. In previous human–animal interaction literature, animals were viewed as catalysts for social interaction (McNicholas & Collis, 2000; Wood et al., 2005), companionship and social connections (Berry et al., 2013; Krause‐Parello et al., 2016; O'Haire, 2013; Winkle et al., 2012). Human–animal connections were also associated with overcoming psychological concerns during task performance, a core participation outcome under the *general tasks and demands* domain (Hu et al., 2018; Hunter et al., 2019; O'Haire, 2013; O'Haire & Rodriguez, 2018; Winkle et al., 2012). As a sense of security and trusting relationships are prominent needs for persons with disability (Hammel et al., 2008), this finding is useful in understanding the value of assistance dog partnerships.

However, the review identified that assistance dog partnerships could also be associated with negative outcomes. These included the financial burden, additional workload to care and train the dog, and the stress associated with integrating the dog into daily life and society. Cost and integration concerns were also identified in other assistance dog reviews (Krause‐Parello et al., 2016; Lippi & Plebani, 2019). Assistance dogs were not always found to have a significant impact on participation for the person living with disability. For example, no study found that assistance dogs had a significant contribution to employment status (O'Haire & Rodriguez, 2018) or capacity to reach items (Vincent et al., 2015), and some had no effect on social outcomes (Shintani et al., 2010) or independence (Rintala et al., 2008; Vincent et al., 2019). This may be attributed to the methodological approaches of included studies or the difficulty in quantifying unique connections between individuals and their dogs.

Assistance dog partnerships could also impact the participation outcomes for the family members of individuals placed with an assistance dog. All participation domains were mapped in relation to outcomes for family members. This is an important finding as health, well‐being and participation in daily life can be impacted by caring for a person with disability (Barzallo, 2018). As persons with disabilities often rely on strong family support systems, the impact of an assistance dog on family members may also indirectly benefit them (Chang & Coster, 2014).

### 4.2 Factors influencing the participation outcomes

This review identified contextual factors that could influence the outcomes of an assistance dog partnership in promoting activity participation (Alcantara et al., 2020). Commonly mapped factors included an individual's mindset, the experience of disability, the social environment, the dog's nature and training, and societal perceptions of assistance dogs. A range of factors specific to the person and their environment need to be considered when matching and placing assistance dogs. Societal understandings of assistance dogs could be a key factor influencing the success of assistance dog placements, and a public lack of knowledge of assistance dogs can impact the support of rights to access for the dog, creating a barrier to community participation (Schoenfeld‐Tacher et al., 2017).

The physical environment was not listed as a limiting factor influencing partnering with an assistance dog, and there were limited data from the review on the influence of the physical environment, such as building design or climate, on the success of assistance dogs. Dogs were described as very adaptable to physical environments, and it is possible that they can help a person to navigate their existing environment rather than needing physical modifications (Lamontagne et al., 2020).

### 4.3 Gaps in assistance dog research

There were several assistance dog training programs and methods of measuring outcomes presented in the review. Some studies lacked detail of the training processes, and training approaches were different between organisations; however, this may be due to programs being commercial in confidence. Future research could be enhanced by the consistent use of ADI definitions and standards (ADI, 2019), including descriptions of the training and purpose of assistance dog placement.

Over 50 standardised tools were used by studies in the review. These tools were not specific to assistance dogs and often were not specific to participation unless mapped to the ICF. Numerous assessment tools were designed by study authors and had no validated psychometric properties. The lack of uniformity between studies is a common concern in animal‐intervention research and poses a challenge when comparing research results. Future research should consider using outcome measures specific to participation while creating and validating outcome measures specific to assistance dog partnerships or creating and evaluating a set of standardised tools. This can promote more consistent approaches to data collection and interpretation. However, a range of tools could also assist in understanding a wide range of outcomes across disability groups or specific disabilities. In this case, the addition of a measure of participation outcomes of the assistance dog partnership could be used.

Studies in the review investigating the outcomes for people living with autism only included children, although participation challenges have been identified by many adults with autism (Burrows & Adams, 2008). Also, only two studies investigating people with diabetes or epilepsy met the inclusion criteria. Many studies were excluded as they only investigated the effectiveness of the dogs in detecting the medical episode, rather than focusing on participation outcomes. The included quantitative studies had study design limitations such as no randomisation, often small sample sizes, and a limited use of participant controls. This may be due to the extensive matching process required for assistance dog placement meaning that random allocation is more ethically challenging for both the participant and the dog.

## 5 STRENGTHS AND LIMITATIONS OF THIS REVIEW

In pursuit of a transparent and rigorous review, this study followed peer‐reviewed scoping review guidelines (Arksey & O'Malley, 2005; Levac et al., 2010). The retrieval of papers was comprehensive as four broad search terms were used across six electronic databases. Evidence was synthesised according to the ICF domains, generating a review with a universally accepted language (WHO, 2001). Additionally, all steps of the process were verified by all authors, strengthening the credibility of findings. However, there is potential that studies were missed as search strategies were limited to papers from peer‐reviewed journals in full‐text English, published in the last 20 years. Also, some studies that may have provided additional information were excluded based on the eligibility criteria. For example, many studies had to be excluded as they were dissertations or because they included guide and hearing dogs where results regarding other assistance dogs could not be extracted.

## 6 CONCLUSION

This scoping review identified how assistance dog partnerships can positively impact on participation, a key factor in promoting quality of life (Magasi et al., 2009). By synthesising the evidence according to the ICF model and domains, key participation outcomes, contextual factors and issues yet to be investigated were identified. Overall, the review is useful in identifying the wide range of participation outcomes that assistance dog partnerships can deliver for people with various disabilities and their families. The impact has yet to be sufficiently acknowledged by funders and policymakers. The review illuminates both potentially desirable and undesired outcomes that need to be considered before engaging in assistance dog partnerships, allowing therapists and clients to weigh the potential pros and cons across the full range of domains and make informed choices about this intervention. For occupational therapists, current findings present a more practical understanding of potential participation outcomes and can allow for better identification of clients who may benefit from a partnership with an assistance dog (Rimmer, 2006). Occupational therapists can use these findings to educate and prepare clients for assistance dog placement—both the desirable and less desirable outcomes and what factors may influence the outcomes—to increase the possibility of success. These findings can also contribute to justification of financing and decision making for applications to funding bodies for provision of an assistance dog which is considered part of an occupational therapy role (Chan et al., 2021). Assistance dog organisations can utilise the understanding of outcomes and the influencing factors when matching dogs and individuals. This comprehensive review has provided tables and information that can guide practitioners seeking to support their recommendations to insurers/payers. For policymakers and funding bodies, this review provides a greater understanding of the potential for positive outcomes for persons with disability and their families. Overall, findings supported that assistance dog partnerships have the potential to align with the objectives of disability services to promote participation.

## FUNDING INFORMATION

This research received no specific grant from any funding agency in the public, commercial or not‐for‐profit sectors.

## CONFLICT OF INTEREST

The authors have no conflict of interest to declare.

## AUTHOR CONTRIBUTIONS

All authors made substantial contributions to the conception and design of the research, the analysis, and interpretation of the data for the review, worked on the drafts and revisions of the work and approved it for publication.

## Supporting information


**Table S1.** Study, participant and assistance dog characteristics with relevant findings

## Data Availability

Data available on request from the authors.
