# Supplementary material for: Understanding the participation outcomes for persons with disability when partnered with assistance dogs: A scoping review
Source: Aust Occup Ther J. 2022 Apr 25;69(4):475–92. doi: 10.1111/1440-1630.12801 (PMC9540062; doi:10.1111/1440-1630.12801)
Supplement: Supplementary file 1 — Table S1. Study, participant and assistance dog characteristics with relevant findings [file AOT-69-475-s001.pdf]

Table 1. Study, participant and assistance dog characteristics with relevant findings

Author, year, origin	Study design; objective	Sample size; disability	Participant demographics	Time with dog (years)	Outcome measures	Key findings related to assistance dog partnerships
**AUTISM (ASD)**						
Burgoyne et al. 2014, Ireland	*Design*: Cross-sectional<br>*Objective*: Safety, public perception, benefits and drawbacks for the person with a disability and their family	*n* =164 (assistance dog = 80, waitlist = 84)<br>*Disability*: ASD	*Age*: r = 4-9<br>*Males*: 89.6%	*NR*	Survey: PCS[i], CGSQ[ii] and questionnaire designed by study. Completed by parent	**Assistance dog v waitlist (single time point):** ↑ parent perceived: safety§, public acceptance§, competency for childcare and management†; – parent strain.<br>**Themes:** Benefits: physical, relationship and family factors; constraints: change, relationship and limiting factors.
(a) Burrows & Adams, 2008a<br>(b) Burrows et al. 2008b<br>Canada	*Design*: Qualitative: ethnography<br>*Objective*: (a) Functional outcomes, (b) Challenges for person with a disability and family	*n* = 10<br>*Disability*: ASD	*Age*: r = 4.5-14<br>*Males*: 70%	*NR*	Interviews of parents, and observations	(a) **Themes:** Public access; community; extra work; seasonality of placement; dog behaviour; maintaining; family factors.<br>(b) **Themes:** Safety; companionship and new skills; shift focus from autism to dog; respite, relaxation, and family recreation; family's social status; facilitating awareness and education about autism.
Fecteau et al. 2016, Canada	*Design*: Non-randomised controlled trial<br>*Objective*: Stress and diurnal cortisol activity for parent	*n* = 98 (experiment = 49, waitlist = 49)<br>*Disability*: ASD, Asperger syndrome, pervasive developmental disorder	*Age*: M = 6.7<br>*Males*: 80%	*Received in study*	PSI-SF[iii], CARS[iv] and saliva samples. Completed by parent	**Experimental (pre - 9 months post placement):** ↓ parental perceptual stress: parent-child interaction‡, total stress†, difficulty of child‡<br>**Experimental v waitlist (pre - 12 weeks post placement):** ↓ cortisol levels: wakening‡, morning§; – cortisol levels: bedtime.
Smyth and Slevin, 2010, Ireland	*Design*: Qualitative: phenomenology<br>*Objective*: Benefits and drawbacks for person with a disability and their family	*n* = 7<br>*Disability*: ASD	*Age*: M = 8.3<br>*Males*: 85.71%	M = 1.75	Interviews of parents	**Themes:** Safety; motor skills; relationships; social support; negative aspects.
Viau et al. 2010, Canada	*Design*: –BA study - single subject design<br>*Objective*: Behaviour and basal salivary cortisol secretion	*n* = 42<br>*Disability*: ASD, Asperger Syndrome, pervasive developmental disorder	*Age*: M = 7.1<br>*Males*: 88.10%	*Received in study*	Saliva samples and questionnaire designed by study. Completed by parent	**Pre v 4 weeks post placement:** ↓ problematic behaviours§; ↓ stress (CAR)‡<br>**With assistance dog v removal of assistance dog**: ↑ stress (CAR)‡<br>**With assistance dog:** – correlation between CAR and average cortisol levels and disruptive behaviours.
**PHYSICAL DISABILITIES**						
Abbud et al. 2014, Canada	*Design*: Case report<br>*Objective*: Mobility and psychosocial	*n* = 2<br>*Disability*: Cerebellar ataxia	*Age*: M = 34.5<br>*Males*: 50%	*NR*	Interviews	**After assistance dog placement: Client A**: Increase participation in education, performance in ambulation, security and social involvement.<br>**Client B**: Improved stair performance, QoL, security, social and retained job.

Author, year, origin	Study design; objective	Sample size; disability	Participant demographics	Time with dog (years)	Outcome measures	Key findings related to assistance dog partnerships
Blanchet et al. 2013, Canada	*Design*: Pre-post *Objective*: Functional mobility	*n* = 34 *Disability*: Spinal, musculo-skeletal injury.	*Age*: M = 45.9 *Males*: 26.5%	*NR*	10-m Walk Test, TUG[v] and stair ascent/ descent test	**Tasks with assistance dog vs without:** ↑ speed for walking†, sit-to-stand‡, speed for stair ascent‡ for (>70.4% of participants); – speed for stair descent.
Camp, 2001, USA	*Design*: Qualitative: ethnography *Objective*: Benefits and drawbacks	*n* = 5 *Disability*: Spinal cord injury, cerebral palsy and lupus, muscular dystrophy, spina bifida, rheumatoid arthritis	*Age*: M = 41.4 *Males*: 60%	M = 11.3	Observations and interviews	**Themes:** Assistance with daily tasks; increased participation; "closer than family"; social acknowledgments; personal skill development; having fun; responsibilities; adjustment period; challenges; independence; someone to watch over me.
Champagne et al. 2016, Canada	*Design*: Pre-post *Objective*: Functional mobility	*n* = 13 *Disability*: Spinal cord injury	*Age*: M = 40.4 *Males*: 76.9%	M = 3.1	Time and rate of perceived exertion on a 630m course	**630m course with assistance dog vs without:** ↓ time taken‡, perceived effort§.
Collins et al. 2006, USA	*Design*: Cross-sectional *Objective*: Psychosocial and community participation	*n* = 152 (assistance dog = 76, control = 76) *Disability*: Spinal cord injury, non-progressive or progressive condition	*Age*: M = 44.4 *Males*: 38.2%	M = 3.1	CES-D[vi], PANAS[vii], RSE[viii], UCLA-3[ix] and CHART[x]	**Assistance dog v control (single time point):** – depressive symptoms, mood, loneliness, self-esteem, positive affect, negative affect. No correlation between length of partnership and community participation or psychosocial well-being.
Crowe et al. 2014, USA	*Design*: Alternating –reatment - single subject design *Objective*: Functional and social in community	*n* = 3 *Disability*: Muscular dystrophy, spinal cord injury, cerebral palsy	*Age*: M = 44.7 *Males*: 0%	M = 4.3	Outcome measures designed by study	**Tasks with assistance dog vs without (6 times in each condition):** Functional tasks: reduced time taken for 4/6 tasks and reduced perceived effort for 5/6 tasks. Social Interaction: increased number of interactions for 2/3 participants and increased satisfaction for 3/3 participants.
Hall et al. 2017, UK*	*Design*: Cross-sectional *Objective*: QoL	*n* = 96 (assistance dog = 72, waitlist = 24) *Disability*: Arthritis, spinal cord injury, multiple sclerosis, stroke, muscular injury/wastage, spinal bifida, spondyloptosis, arthrogryposis, cerebral palsy, neurological disorders, dystonia, scleroderma, ehlers-danlos syndromes, other	*Age*: 18+ *Males*: 19.8%	*NR*	Survey: Adapted Flanagan Quality of Life scale[xi]	**Assistance dog v waitlist (single time point):** ↑ overall QoL§, material comforts†, health‡, relationship with relatives†, volunteering§, participating in organisations‡, learning†, understanding of one's self‡, participation in work†, creative expression‡, socialising§, active recreation†, reading/music§, independence§; – having or rearing children, relationship with spouse or close friends.
Herlache-Pretzer et al. 2017, USA	*Design*: Qualitative: descriptive *Objective*: Occupational engagement	*n* = 4 *Disability*: Mobility impairment	*Age*: M = 42 *Males*: 0%	M = 4.75	Interviews	**Themes:** Assistance dog as a form of assistive technology; community integration (positives and challenges); comparing to other forms of assistive technology (adaptability, extra effort and expense, fluctuation); relationship with assistance dog.

**Author, year, origin**	**Study design; objective**	**Sample size; disability**	**Participant demographics**	**Time with dog (years)**	**Outcome measures**	**Key findings related to assistance dog partnerships**
Hubert et al.<br>2013,<br>Canada	*Design*: Pre-post<br>*Objective***:** Mobility, pain, social participation and QoL	*n* = 11<br>*Disability*: Spinal cord injury	*Age*: M = 32.7<br>*Males*: 76.9%	*Received in study*	WUSPI[xii], LIFE-H[xiii], QLI[xiv], RNLI[xv], WST[xvi] 12-MWT[xvii], exertion scale	**Assistance–dog (pre - 7 months post placement):** ↑ social participation†, return to normal life†, wheelchair skills†, endurance† effort†; ↓ shoulder pain during daily tasks‡; – QoL.<br>**Use of wheelchair without assistance–dog (pre - 7 months post placement):** ↓ effort†; – wheelchair skills, endurance.
Lamontagne et al.<br>2019,<br>Canada*	*Design*: Qualitative: phenomenology<br>*Objective***:** Facilitators and barriers to functional outcomes	*n* = 9<br>*Disability*: Spinal cord injury, cerebral palsy, congeal malformation, scoliosis, polytrauma	*Age*: NR<br>*Males*: 55.6%	1.5+	Interviews with 9 people with disabilities and 13 rehabilitation professionals	**Themes:** Facilitators/barriers: knowledge, beliefs about capabilities, beliefs about consequences, environmental factors, role and identity; function: daily tasks, social participation, positive emotions, friendship.
(a) Lundqvist et al. 2018, Sweden*<br>(b) Lundqvist et al. 2019, Sweden*	*Design*: Pre-post<br>*Objective***:**<br>(a) QoL, activity-level and well-being<br>(b) Cost effectiveness	*n* = 30<br>*Disability*: Neuromusculo, musculo-skeletal, other	*Age*: *NR*<br>*Males*: *NR*	*Received in study*	(a) EQ-5D single index[xviii], EQ-VAS and SF-6D (RAND-36)[xix]<br>(b) Cost effectiveness analysis in relation to quality-adjusted life years, using Markov model	**(a) Assistance dog (pre – 3 months post placement):** ↑ physical role functioning‡, emotional role functioning†, health transition score†; – health related QoL, physical, bodily pain, general health, vitality, social and mental health.<br>**(b) Assistance dog v companion dog:** Assistance dog was associated with a 52% decrease in overall costs and an 87% gain in quality of life years Probability of cost effectiveness increased as the cost threshold increased.
Rintala et al.<br>2008,<br>USA*	*Design*: Pre-post<br>*Objective***:** Functional tasks and satisfaction	*n* = 31 (control only = 13, experiment only = 16, both = 2)<br>*Disability*: Mobility impairment	*Age*: M = 49.2 (experiment), M = 45.1 (control)<br>*Males*: 22.2% (experiment), 26.7% (control)	*Received in study*	12-SF[xx], FIM[xxi], CHART[xxii], SLS[xxiii] and outcome measures designed by study	**Experiment (pre – 6 months post placement) and experiment v control (6 months post placement):** – physical and mental health impact on tasks, task assistance, life satisfaction, independence, mobility, occupations.<br>**Experiment (pre – 6 months post placement):** ↓ paid assistance<br>**Experiment reported (6 months post placement):** Life satisfaction (M = 8.94/10), positive impact on ADL (78%), emotional support/companionship (44%), negative aspects (61%), desired additional tasks (44%), at least one less assistive device used (33%).
Shintani et al.<br>2010,<br>Japan	*Design*: Cross-sectional<br>*Objective***:** QoL	*n* = 38 (assistance dog = 10, control = 28)<br>*Disability*: Spinal cord injury, multiple sclerosis, rheumatoid arthritis, myasthenia gravis, cerebral haemorrhage, stroke, spino-cerebellar degeneration, lupus	*Age*: M = 48.5<br>*Males*: 44.7%	M = 1.7	Survey: SF-36[xxiv]	**Assistance dog v control (single time point):** ↑ physical functioning†, role emotional† overall mental health‡; – role physical, vitality, social functioning, mental health, overall physical, bodily pain and general health.

Author, year, origin	Study design; objective	Sample size; disability	Participant demographics	Time with dog (years)	Outcome measures	Key findings related to assistance dog partnerships
Vincent et al. 2015, Canada	*Design*: Pre-post<br>*Objective*: Functional mobility and tasks	*n* = 199<br>*Disability*: Spinal musculo-skeletal, central or peripheral nervous system injury	*Age*: M = 43.1<br>*Males*: 52%	*r* = 2-4	Outcome measure designed by study: mobility (flat terrain, slope, curb) and picking up objects.	**Mobility with assistance dog vs without:** ↑ distance and speed travelled on flat terrain§, performance ascending slope and threshold – performance descending slope and threshold.<br>**Picking up items with assistance dog v without:** 15/51 (29%) improved compared to a long-handed-reacher and 57/129 (44%) improved compared to no assistive device.
Vincent et al. 2019, Canada	*Design*: Longitudinal study with repeated measure<br>*Objective*: Pain, fatigue, functional tasks and satisfaction	*n* = 17<br>*Disability*: Spinal cord injury, cerebral palsy, traumatic leg amputation, spastic diplegia	*Age*: M = 41.9<br>*Males*: 52.9%	*Received in study*	WUSPI[12], RPE[xxv], SF-36[xxvi], WST[16], WST-Q[xxvii] , Jamar dynamometer, COPM[xxviii], RNLI[15], LSA[xxix], PIADS[xxx] and QUEST 2.0[xxxi]	**Pre v 3 months v 6 months v 9 months post placement:** ↓ pain‡, rate of perceived exertion‡; ↑ satisfaction of activities of normal life†, vitality: full of life†, wheelchair: steep slopes§, wheelchair: soft surfaces§, satisfaction of personal goals‡, mobility§, work†, leisure‡; – energy, worn out, grip strength, space mobility, wheelchair: threshold.<br>**3 months v 6 months v 9 months post placement:** High and constant satisfaction with the psychosocial impact of the dog
White et al. 2017, UK	*Design*: Cross-sectional<br>*Objective*: Pet attachment impact on QoL	*n* = 73<br>*Disability*: Physical disabilities^	*Age*: 18+<br>*Males*: 23.2%	M = 4.6	Survey: PAQ[xxxii] and QoLs[xxxiii]	**Assistance dog:** anxious attachment to assistance dog is a predictor of QoL†.
			**MENTAL HEALTH**			
Crowe et al. 2018a, USA	*Design*: Qualitative: exploratory<br>*Objective*: Emotional and physical well-being and interactions in the home	*n* = 6<br>*Disability*: Veterans with PTSD, or TBI and PTSD	*Age*: M = 43.3<br>*Males*: 66.7%	≥1	Interviews	**Themes:** Providing physical safety and peace of mind; supporting healthy behaviours; "my hero"; influencing family and friend relationships; challenges.<br>**Overarching theme:** Improving occupational performance in the home.
Crowe et al. 2018b, USA	*Design*: Qualitative: exploratory<br>*Objective*: Benefits, drawbacks and emotional and physical well-being	*n* = 9<br>*Disability*: Veterans with PTSD and/or TBI	*Age*: M = 35.8<br>*Males*: 100%	M = 3.4	Focus groups and interviews	**Themes:** Secluded by seeking society; opening opportunities; bridging the gap; reclaiming life.<br>**Overarching theme:** Calming catalyst.
Husband et al. 2020, Canada	*Design*: Exploratory case study<br>*Objective*: Substance-use and well-being	*n* = 4<br>*Disability*: PTSD	*Age*: *NR*<br>*Males*: 75%	≈2 years	Interviews and medication history	**All participants reported:** Reduced PTSD symptoms, increased feeling of support, increased task participation and reduction/stabilisation of medication intake.
Krause-Parello & Morales. 2018, USA	*Design*: Qualitative: phenomenology<br>*Objective*: Lived experience	*n* = 21<br>*Disability*: Veterans with mental illness	*Age*: M = 44.6<br>*Males*: 66.7%	M = 2.0	Interviews	**Themes:** Psychosocial functioning; value: emotional, therapeutic, familial; detriments: public ignorance about disability, financial and time encumbrance.

Author, year, origin	Study design; objective	Sample size; disability	Participant demographics	Time with dog (years)	Outcome measures	Key findings related to assistance dog partnerships
Lessard et al. 2018, Canada	*Design*: Qualitative: case series<br>*Objective*: PTSD management	*n* = 10<br>*Disability*: Veterans with PTSD	*Age*: 31+<br>*Males*: 90%	r = 2-4	Interviews	**Themes:** Assistance dog's roles and tasks; usability: companionship, medication intake, outing and physical activities, pre-existing social network, security bubble, social interactions and relationships, symptoms, therapeutic process; obstacles: dogs health, employee'', public's and relatives' reactions, maintenance.
Lloyd et al. 2019, Australia	*Design*: Cross-sectional<br>*Objective*: Functional outcomes and relationship	*n* = 199<br>*Disability*: Mental illnesses	*Age*: median = 47<br>*Males*: 33%	*NR*	Online survey designed by study	**Assistance dog:** Mixed impact on use of healthcare and mental-health services.<br>**Themes:** Independence; confidence; social function; companionship; safety; hope. 198/199 had a positive partnership.
McLaughlin & Hamilton. 2019, Australia	*Design*: Qualitative<br>*Objective*: Occupational outcomes	*n* = 7<br>*Disability*: Veterans with PTSD	*Age*: *NR*<br>*Males*: 85.7%	r = 0.4-3	Focus groups	**Themes:** Isolation; safety; lifeline; self-management; reconnection; challenges.
O'Haire & Rodriguez. 2018, USA	*Design*: Longitudinal study<br>*Objective*: Mental health, QoL, social function and employment	*n* = 141 (assistance dog = 75, waitlist = 66)<br>*Disability*: Veterans with PTSD	*Age*: M = 37.1<br>*Males*: 78.0%	M = 1.6	PHQ-9[xxxiv], BSPW[xxxv], PROMIS[xxxvi], ALS[xxxvii], WPAI[xxxviii], PCL[xxxix], SLS[23], CDRS[xl], VR-12[xli]	**Assistance dog v waitlist (single time point):** ↑ perceived improvement from treatment‡, QoL‡, social functioning†, work participation†, companionship†, mental health function§; ↓ PTSD and depressive symptoms§, activity impairment† social isolation§; – physical, employment.<br>**Assistance dog (pre v 3 weeks post v follow up):** ↓ PTSD symptoms§
Rodriguez et al. 2018, USA	*Design*: Cross-sectional<br>*Objective*: Cortisol awakening response and mental health functioning	*n* = 73 (assistance dog = 45, waitlist = 28)<br>*Disability*: Veterans with PTSD (some with TBI)	*Age*: M = 37<br>*Males*: 80.8%	M= 1.7	Saliva samples, survey: medication use, PCL[39], VR-12[xlii] PROMIS[xliii] and PSQI[xliv]	**Assistance dog v waitlist (single time point):** ↓ anxiety§, anger‡, sleep disturbance‡, symptoms of alcohol abuse†, Cortisol levels AUCi (area under the curve)†, and CAR (cortisol awakening response)†; – sleep quality.<br>**Assistance dog:** Positive correlation between improvements in sleep disturbance and length of partnership.
Vincent et al. 2017, Canada	*Design*: Longitudinal study<br>*Objective*: QoL, mental health and daily and social function	*n* = 15<br>*Disability*: Veterans with PTSD	*Age*: 31+<br>*Males*: 75%	*Received in study*	PCQI[44], PCL-M[xlv], BDI-II[xlvi], LSA[29], WHOQOL BREF[xlvii]	**6 months pre v 3 months pre v placement v 3 months post placement:** ↑ sleep quality‡ QoL (social domain)†; ↓ PTSD symptoms§, depression symptoms†; – mobility in home/community, QoL
Yarborough et al. 2017, USA	*Design*: Mixed methods<br>*Objective*: Functional outcomes	*n* = 78 (assistance dog = 24, waitlist = 54, but 22 received assistance dog in study)<br>*Disability*: Veterans with PTSD	*Age*: M = 42.4<br>*Males*: 67.9%	Assistance dog: M = 2.2	VR-12[41], PCL-C[xlviii], DRRI[xlix], BASIS-24[l], GSS[li], Wisconsin Quality of Life Index, and designed by study	**Assistance v waitlist (single time point):** ↑ mental health‡, interpersonal relationship†, happiness‡, QoL‡,↓depression§, PTSD symptoms‡; – physical, psychosis, activity level, substance abuse, emotional lability.<br>**Waitlist v received assistance dog:** ↓ depression†, emotional lability‡, PTSD symptoms‡; ↑ mental health‡, activity level‡, happiness†, QoL‡; – substance abuse, psychosis, physical and interpersonal relationship.

Author, year, origin	Study design; objective	Sample size; disability	Participant demographics	Time with dog (years)	Outcome measures	Key findings related to assistance dog partnerships
Yarborough et al.<br>2018,<br>USA	*Design*: Qualitative<br>*Objective*: Benefits and drawbacks	*n* = 41 (assistance dog = 22, received assistance dog = 19)<br>*Disability*: Veterans with PTSD	*Age*: M = 45<br>*Males*: 71%	*Received in study* (19)<br>M = 2.2 (22)	Interviews and observations with 41 people with disability, 8 carers and 6 trainers	**Themes:** Benefits**:** reduce hypervigilance, improving sleep quality and duration, remain present, reconnection with humans, improvements beyond PTSD symptoms; challenges**:** preparations, pressure to learn multiple commands, difficult for caregiver to allow the dog to assist, dog in the community can be overwhelming for partner.
**DISABILITY ALERT**						
(a) Lundqvist et al.<br>2018,<br>Sweden***<br>(b) Lundqvist et al.<br>2019,<br>Sweden***	*Design*: Pre-post<br>*Objective***:**<br>(a) QoL, occupations and well-being<br>(b) Cost effectiveness	*n* = 20<br>*Disability*: Diabetes	*Age*: *NR*<br>*Males*: *NR*	*Received in study*	(a) EQ-5D single index[18], EQ-VAS and SF-6D (RAND-36)[19]<br>(b) Cost-effectiveness analysis in relation to quality-adjusted life years, using Markov model	(a) **Assistance dog (pre-post):** – health related QoL, physical role, emotional role, health transition score, physical functioning, bodily pain, general health, vitality, social and mental health.<br>**(b) Assistance dog v companion dog:** In the simulation, an assistance dog associated with 54% decrease in overall costs (decrease in health care costs, informal care, productivity loss (due to sick leave), municipal services). Associated with an 55% gain in quality of life years. Probability of cost effectiveness increased as the cost threshold increased.
Plowman et al.<br>2009,<br>Australia	Qualitative Case study<br>*Objective***:** Well-being for person living with a disability and their parent	*n* = 1<br>*Disability*: Epilepsy	*Age*: *13*<br>*Males*: *100%*	1	Interviews with person with a disability and parent	**Themes:** Life around epilepsy; the support of an assistance dog; the influence of an assistance dog.
**COMBINATION**						
Bibbo et al.<br>2019,<br>USA	*Design*: Cross-sectional<br>*Objective***:** Psychosocial, QoL and function for family members	*n* = 128 (assistance dog = 77, waitlist = 51)<br>*Disability*: Seizure condition, musculoskeletal, neuromuscular, developmental	*Age*: M = 22.2<br>*Males*: 44.5%	M = 4.7	PedsQL[lii], PROMIS[liii] and MDORS[liv]. Completed family members.	**Assistance dog v waitlist (single time point):** ↑ psychosocial health†, emotional functioning†, family relationships†; ↓ worry†; – social, physical, cognitive, family, work/school, anger, companionship, sleep, QoL, communication, daily activity.
Gravrok et al.<br>2019a<br>Australia**	*Design*: Qualitative case study<br>*Objective***:** Drawbacks	*n* = 5<br>*Disability*: Seizures, mental illness, mobility, other	*Age*: 12+<br>*Males*: 60%–	r ≈ 0.5 - 1	Interviews with 5 people with disability, 4 parents, 4 instructors, 1 carer, and 1 psychologist	**Themes:** Medical condition; cognitive ability; social environment; dog factors

Author, year, origin	Study design; objective	Sample size; disability	Participant demographics	Time with dog (years)	Outcome measures	Key findings related to assistance dog partnerships
Gravrok et al. 2019b, Australia**	*Design*: Qualitative case study<br>*Objective*: Benefits and drawbacks	*n* = 6<br>*Disability*: Seizures, mental illness, mobility, other	*Age*: M = 24.5<br>*Males*: 66.7%	≈ 1 month	Interviews with 5 people with disability and 5 parents.	**Themes:** Initial response: intensity of change, routine, rules; renegotiating relationships and social interactions: family, public reaction, parent role, unnecessary assistance, lack of understanding by community; adjusting expectations regarding the assistance dog's behaviour and capabilities.
(a) Rodriguez et al. 2020a, USA<br>(b) Rodriguez et al. 2020b, USA.	*Design*: Cross-sectional<br>*Objective*:<br>(a) Psychosocial and well-being<br>(b) Physical, psychosocial, benefits and drawbacks	*n* =<br>(a) 154 (assistance dog = 97, waitlist = 57)<br>(b) 91/154 (assistance dog = 64, waitlist = 27)<br>*Disability*: Seizure, 7usculos, musculo-skeletal neuro-muscular developmentalknown	(a)<br>*Age*: M = 26.3<br>*M*: 53%<br>(b)<br>*Age*: M = 34.8<br>*Males*: 47%	(a) *NR*<br>(b) M = 5	Survey:<br>(a) PedsQL[lv] PROMIS[lvi] and MDORS[lvii] completed by persons with disability or parent<br>(b) questions designed for study	**(a) Assistance dog v waitlist (single time point):** ↑ psychosocial§, emotional functioning‡, social functioning†, work/school functioning§; – anger, companionship, sleep disturbance.<br>**Assistance dog:** Positive correlation between time with assistance dog and psychosocial†. High scores for human-animal bond.<br>**(b) Themes:** Benefits: physical (medical assistance, physical assistance), psychosocial (human animal relationship, emotional, QoL, social); drawbacks: dog care, public access and education, lifestyle adjustment, dog behaviour, other.

Key: † = p<0.05; ‡ = p<0.01; § = p< 0.001; ↑ = greater/improved; ↓ = less/reduced; **-** = minimal/no change/lack of significant finding; * = only data including people with physical disability was extracted; ** = data relating guide dogs was not extracted; *** = only data including people with diabetes was extracted; ^ = extraction determined through correspondence with study authors; ≈ = approximately; ABA = comparison, treatment, comparison study design; ASD = autism spectrum disorder; M = mean; NR = not reported, PTSD = post-traumatic stress disorder; QoL = quality of life; r = range; TBI = traumatic brain injury
Note: Endnotes for the full names of the outcome measures and subscales used can be found in Appendix 7.

[i] Perceived Competence Scales
[ii] Caregiver Strain Questionnaire
[iii] Parenting Stress Index – short form
[iv] Childhood Autism Rating Scale
[v] Timed "up & go" test
[vi] Centre for Epidemiologic Studies – Depression Scale
[vii] Positive and Negative Affect Scale (*positive affect* and *negative affect* subscales)
[viii] Rosenberg Self-Esteem Scale
[ix] UCLA Loneliness Scale – Version 3
[x] Craig Handicap and Assessment Reporting Technique
[xi] Flanagan Quality of Life Scale (QOLS). Adapted to include an additional item on independence.
[xii] Wheelchair User's Shoulder Pain Index
[xiii] Assessment of Life Habits
[xiv] Quality of Life Index
[xv] Reintegration to Normal Living Index
[xvi] Wheelchair Skills Test

[xvii] 12-minute walk test
[xviii] EuroQol- 5 Dimension
[xix] Health State Short Form derived from the Short-Form 36 Health Survey (SF-36)
[xx] 12 Item Short Form Health Survey
[xxi] Functional Independence Measure – Motor Scale
[xxii] The Craig Handicap Assessment and Reporting Technique (*physical*, *independence*, *mobility* and *occupation* subscales)
[xxiii] Satisfaction with Life Scale
[xxiv] 36 Item Short-Form Health Survey
[xxv] Rate of Perceived Exertion scale
[xxvi] 36 Item Short-Form Health Survey (*vitality* subscale)
[xxvii] Wheelchair Skills Test Questionnaire
[xxviii] Canadian Occupational Performance Measure
[xxix] Life Space Assessment
[xxx] Psychosocial Impact of Assistive Devices Scale
[xxxi] Quebec User Evaluation of Satisfaction with Assistive Technology
[xxxii] The Pet Attachment Questionnaire
[xxxiii] The Quality of Life Scale
[xxxiv] The Patient Health Questionnaire
[xxxv] Bradburn Scale of Psychological Well-being
[xxxvi] The Patient-Reported Outcomes Measurement Information System – Depression adult short-form, Ability to Participate in Social Activities adult short, Social Isolation adult short, Companionship adult short form
[xxxvii] The American Legion Survey of Patient Healthcare Experiences
[xxxviii] Work Productivity and Activity Impairment Questionnaire: General Health – Version 2.0
[xxxix] The PTSD Checklist
[xl] Connor Davidson Resilience Scale
[xli] Veteran's RAND 12-Item Health Survey
[xlii] Veteran's RAND 12-Item Health Survey (*physical component score*)
[xliii] Patient-Reported outcome measurement information system – Anxiety (8A) adult short form, Anger (5A) adult short form, Alcohol Use (7A) adult short form, and Sleep Disturbance (8A) adult short form
[xliv] Pittsburgh Sleep Quality Index.
[xlv] Posttraumatic Stress Disorder Checklist for Military
[xlvi] Beck Depression Inventory.
[xlvii] Brief World Health Organisation Quality of Life Questionnaire.
[xlviii] Posttraumatic Stress Disorder Checklist for Civilians
[xlix] Deployment Risk and Resilience Inventory–2.
[l] Behaviour and Symptom Identification Scale.
[li] General Social Survey
[lii] The Pediatric Quality of Life Inventory - Adult Quality of Life Inventory Version 4.0 Short Form. (*emotional functioning*, *social functioning* and *work/school functioning* subscales.)
[liii] Patient-Reported Outcomes Measurement Information System – Anger (5A) adult short form, Companionship (4A) short form 4a and Sleep Disturbance (4A) short form
[liv] Monash Dog Owner Relationship Scale (*perceived emotional closeness* and *dog–owner interaction* subscales)
[lv] Pediatric Quality of Life Inventory 4.0 - Generic Core Scales (*emotional functioning*, *social functioning* and *work/school functioning* subscales)

[lvi]The Patient-Reported Out- comes Measurement Information System – Anger (5A) short form, Companionship (4A) short form and Sleep Disturbance (4A) short form
[lvii] Monash Dog Owner Relationship Scale (*emotional closeness*, *dog–owner interaction*, and *perceived costs* subscales)
